# IL-6 receptor antibody treatment improves muscle weakness in experimental autoimmune myasthenia gravis mouse model

**DOI:** 10.3389/fneur.2024.1356300

**Published:** 2024-05-01

**Authors:** Shota Miyake, Kenichi Serizawa, Shinichi Onishi, Yoshichika Katsura, Masayuki Baba, Mitsue Kurasawa, Haruna Tomizawa-Shinohara, Keigo Yorozu, Yoshihiro Matsumoto, Mariko Noguchi-Sasaki

**Affiliations:** Product Research Department, Chugai Pharmaceutical Co., Ltd., Yokohama, Japan

**Keywords:** myasthenia gravis, IL-6, IL-6R antibody, acetylcholine receptor, follicular helper T cells

## Abstract

Myasthenia gravis (MG) is a chronic autoimmune disease characterized by muscle weakness and fatigue. It is caused by pathological autoantibodies against components expressed at neuromuscular junctions, such as acetylcholine receptor (AChR). Interleukin-6 (IL-6) has been suggested to play a role in the pathogenesis of MG, and IL-6 receptor (IL-6R) antibody treatment may provide a novel therapeutic option. In this study, we investigated the effects of IL-6R antibody treatment in an experimental autoimmune MG (EAMG) mouse model. We demonstrated that IL-6R antibody treatment improved muscle weakness, reduced IgG deposition at neuromuscular junctions, and the levels of AChR autoantibodies in serum. In addition, follicular helper T cells and Th17, plasma cells in lymph nodes were lower in IL-6R antibody treated mice. Our findings suggest that IL-6R blockade may be a novel and effective therapeutic strategy for the treatment of MG.

## 1 Introduction

Myasthenia gravis (MG) is an autoimmune disease caused mainly by pathological autoantibodies against components expressed at neuromuscular junctions, such as acetylcholine receptor (AChR) and muscle-specific tyrosine kinase (MuSK) (1). Myasthenia gravis (MG) is categorized into two primary subtypes: generalized myasthenia gravis (gMG), which predominantly involves the limbs and various ocular muscles, and ocular myasthenia gravis, which is characterized by the selective involvement of the extraocular muscles (2). Approximately 80% of MG patients have AChR autoantibodies (1). AChR autoantibodies affect neuromuscular signaling via at least three mechanisms: first is by binding to AChR and causing complement-dependent cytotoxicity at neuromuscular junctions; second is by antibody-induced crosslinking of AChR, which induces endocytosis and subsequently leads to the degradation of the receptors; and third is by functionally blocking AChR (3). As a result, MG is characterized by generalized muscle weakness accompanied by fatigue and fluctuating symptoms (e.g., ptosis, diplopia, dysarthria, dysphagia, muscle weakness in the extremities and neck, and respiratory muscle paresis).

Traditional therapies for gMG include corticosteroids and other immunosuppressants, thymectomy, plasmapheresis, and intravenous immunoglobulin. In addition to these, new molecularly biological therapeutics, such as C5 inhibitors and neonatal Fc receptor (FcRn) inhibitors, have been developed recently. Although these biologics are efficacious for some gMG patients, about 30–40% of gMG patients fail to respond to any existing treatments and hence an unmet need remains (4–8).

Interleukin-6 (IL-6) is a pleiotropic cytokine involved in the pathogenesis of several autoimmune diseases, and the relationship between IL-6 and gMG pathogenesis has been also explored in various studies. In one study, serum IL-6 levels were found to be significantly elevated in gMG patients compared to healthy volunteers, and those levels were positively correlated with the severity of the disease (9). That study also found that IL-6 levels decreased after immunosuppressive therapy for MG, suggesting a potential role of IL-6 in MG pathogenesis (9). IL-6 is known to exert pleiotropic biological effects such as activation and differentiation of B and T cells. IL-6 is reported to be involved in differentiation of follicular helper T cells (T_FH_ cells) and survival of plasmablasts and to promote production of autoantibodies (10–12). T_FH_ and B cells are known to play significant roles in the production of pathogenic autoantibodies in gMG (13), and circulating T_FH_ cells (cT_FH_ cells) are associated with severity of gMG (14). In addition, IL-6 is known to regulate the balance between IL-17 producing Th17 and regulatory T (Treg) cells (15). An imbalance in Th17 and Treg has been observed in MG (16, 17), suggesting that a disruption in the Th17/Treg balance could contribute to the onset of disease. Therefore, inhibition of IL-6 signaling might be a potential therapeutic strategy for gMG patients.

In this study, we investigated the efficacy and potential mechanisms of action of IL-6 receptor (IL-6R) antibody treatment in an AChR-immunized experimental autoimmune myasthenia gravis (EAMG) mouse model, an animal model that exhibits immunopathological features closely mimicking human myasthenia gravis, including muscle weakness and AChR autoantibodies in serum (18).

## 2 Materials and methods

### 2.1 Animals

Female C57BL/6J mice (8 weeks old; Jackson Laboratories Japan, Inc., Kanagawa, Japan) were used. All mice were fed ordinary laboratory chow and allowed free access to water under a consistent 12-h light/dark cycle. All animal procedures were conducted in accordance with the Guidelines for the Care and Use of Laboratory Animals at Chugai Pharmaceutical Co., Ltd., and all experimental protocols were approved by the Animal Care Committee of the institution and conformed to the Guide for the Care and Use of Laboratory Animals published by the US National Institutes of Health.

### 2.2 Experimental design of EAMG mouse model

Torpedo AChR was purified from electroplaque tissue of *Torpedo californica* as described in Wu et al. (19), with minor modifications. On Day 0, mice were immunized by subcutaneous inoculation at a hind footpad and a shoulder with 100 μg of the purified AChR emulsified in complete Freund’s adjuvant (CFA) supplemented with *Mycobacterium tuberculosis* extract H37Ra (231141; Becton Dickinson, Franklin Lakes, NJ, United States). Control mice were treated with complete Freund’s adjuvant and phosphate-buffered saline (PBS) alone. Four weeks after the initial inoculation, the mice received a booster inoculation at a shoulder and the base of the tail with the same amount of AChR in incomplete Freund’s adjuvant (263910; Becton Dickinson). The control mice received a similar booster without AChR. Muscle strength was evaluated for 8 weeks after the first inoculation once a week. And tibialis anterior muscle, serum and inguinal lymph nodes were harvested for immunohistochemistry, electrochemiluminescence immunoassay, real-time PCR analysis 4 weeks after booster inoculation. On 1 week after booster inoculation, inguinal lymph nodes were harvested for flow cytometric analysis.

### 2.3 Drugs

Rat anti-mouse IL-6 receptor antibody (MR16-1) was prepared using a hybridoma established in our laboratory (17). Mice were intraperitoneally administered MR16-1 (8 mg/mouse) or vehicle (PBS) just before the first and the booster inoculation. In addition, the mice were administered MR16-1 (0.5 mg/mouse) or vehicle once a week, excluding the immunization weeks, for the duration of the experiment.

### 2.4 Grip strength assessment

Grip strength assessment was conducted in principle as described in Mori et al. (20). Forelimb muscle strength was measured with an MK-380CM/R grip-strength meter (Muromachi Kikai, Tokyo, Japan). Grip strength was evaluated after mice had made 30 consecutive paw grips to the top of their steel grid cage. Seven measurements were performed per mouse; five of the seven measurements (excluding the maximum and minimum) were used for statistical analysis.

### 2.5 Immunohistochemistry

Frozen slices of tibialis anterior muscle were prepared as follows. Mice were euthanized under anesthesia with isoflurane, and their tibialis anterior muscles were removed. Samples were embedded in optimal cutting temperature compound (45833; Sakura Finetek, Tokyo, Japan). Slices were fixed with 4% paraformaldehyde (09154-85; Nacalai Tesque Inc., Kyoto, Japan) for 10 min at room temperature and then stained by overnight incubation at 4°C with alpha-bungarotoxin conjugated to Alexa Fluor 555 (B35451; Thermo Fisher Scientific, Waltham, MA, United States) and with goat anti-mouse IgG H&L conjugated to Alexa Fluor 488 (ab150117; Abcam, Cambridge, United States).

Whole-slide images were obtained with a NanoZoomer S60 digital slide scanner (Hamamatsu Photonics, Shizuoka, Japan). Mouse IgG-positive and AChR-positive areas were quantified by using HALO AI imaging analysis software (v3.4.2986.209; Indica labs, NM, United States).

### 2.6 Electrochemiluminescence immunoassay

Mice were anesthetized with isoflurane and blood was collected from the inferior vena cava. Electrochemiluminescence (ECL) assay for the detection of anti-mouse AChR autoantibodies was conducted as follows. Mouse AChR was purified from the muscle of mice that had been used in other studies. Biotinylated alpha-bungarotoxin (B1196; Thermo Fisher Scientific) was immobilized onto 96-well streptavidin-coated plates (L15SA-1; Meso Scale Discovery, Rockville, MD, United States) by incubating for 1 h at room temperature. The plates were then washed 3 times with PBS-T (T9183; Takara Bio Inc., Shiga, Japan). After washing, purified mouse AChR was added to each well and incubated for 1 h at room temperature. After 3 washes, diluted serum was added to each well and incubated for 1 h at room temperature. After 3 washes, SULFO-TAG labeled goat anti mouse IgG H&L (R32AC-1; Meso Scale Discovery) was added and incubated for 1 h at room temperature. Staining with the following secondary antibodies was used to measure IgG isotypes: sulfo-tag conjugated goat anti-mouse IgG1 (D22JL-2; Meso Scale Discovery), sulfo-tag conjugated goat anti-mouse IgG2a (D22JM-2; Meso Scale Discovery), sulfo-tag conjugated goat anti-mouse IgG2b (D22JN-2; Meso Scale Discovery), and sulfo-tag conjugated goat anti-mouse IgG3 (D22JO-2; Meso Scale Discovery). After washing, Read Buffer T (R92TC-1; Meso Scale Discovery) was added. Light intensity was measured with a MESO QuickPlex SQ 120MM Reader (Meso Scale Discovery).

### 2.7 Flow cytometric analysis

Lymph nodes were passed through 40 μm cell strainers to obtain single cell suspensions. For Th1 and Th17 analysis, cells were stimulated for 4 h in RPMI 1640 (R8758; SIGMA, St. Louis, MO, United States) containing 10% FBS (F2442; SIGMA), 55 μM 2-Mercaptoethanol (21985–023; Thermo Fisher Scientific), 10 mM HEPES (H0887; SIGMA), 1 mM Sodium pyruvate (11360–070; Thermo Fisher Scientific) and 100 U /ml Penicillin–Streptomycin (15140-122; Thermo Fisher Scientific) with cell stimulation cocktail containing phorbol 12-myristate 13-acetate and ionomycin (00-4970; Thermo Fisher Scientific) in the presence of 0.1% of BD GolgiPlug (555028; Becton Dickinson). Mononuclear cells were incubated with Mouse BD Fc Block (553142; Becton Dickinson) before staining. The cells were initially stained with the antibody of FITC-conjugated anti-CD4 (100510, BioLegend, San Diego, CA, United States), and then intracellular stained using the antibodies of PE-conjugated anti-IL-17A (506904, BioLegend), and APC-conjugated anti-IFN-γ (505810, BioLegend) performed with the Fixation/Permeabilization Solution Kit with BD GolgiPlug (555028; Becton Dickinson) according to the manufacturer’s protocol. For T_FH_ and Treg cell analysis, mononuclear cells were incubated with Mouse BD Fc Block before staining. The cells stained with the antibodies of FITC conjugated anti-CD4, biotinylated anti-CXCR5 (13-7185-82; Thermo Fisher Scientific), BV786-conjugated anti-CD25 (564023; Becton Dickinson), PE/Cyanine7 conjugated anti PD-1 (109110; BioLegend), and then APC-streptavidin (405207; BioLegend) was utilized to detect biotinylated primary antibody. Intracellular stained using the antibodies of PE-conjugated anti Foxp3 (12-5773-80B; Thermo Fisher Scientific) performed with the Anti-Mouse/Rat Foxp3 Staining Set (72-5775-40; Thermo Fisher Scientific) according to the manufacturer’s protocol. For B and plasma cells analysis, mononuclear cells were incubated with Mouse BD Fc Block before staining. The cells were stained with the antibody of FITC conjugated anti-B220 (553088; Becton Dickinson), BV605 conjugated anti-CD19 (115540; BioLegend), APC conjugated anti-CD138 (558626; Becton Dickinson), PE conjugated anti-TACI (133403, BioLegend). Data were acquired using BD LSRFortessa X-20 (Becton Dickinson) and analyzed using FlowJo 10.5.3 (FlowJo, Ashland, OR, United States).

### 2.8 Real-time PCR analysis

Total RNA was isolated from the inguinal lymph nodes by using an RNeasy mini kit (74106; Qiagen, Venlo, Netherlands). TaqMan quantitative real-time PCR (qRT-PCR) was performed with TaqMan Gene Expression Assays for *Il21* (Mm00517640_m1), *Bcl6* (Mm00477633_m1), *Prdm1* (Mm00476128_m1), *Irf4* (Mm00516431_m1), and *Xbp1* (Mm00457357_m1) in QuantStudio 7 Pro (Thermo Fisher Scientific). Gene expression was normalized to the endogenous control, GAPDH (4352661; Thermo Fisher Scientific).

### 2.9 Statistical analyses

All data are expressed as mean and SD. The *n* values refer to the number of individual animals in each group on which experiments were performed. The statistical significance of differences was determined by using Tukey’s multiple comparison test or Steel–Dwass’s multiple comparison test for comparison of multiple groups. Two-way ANOVA was used for comparison of time course data. Statistical significance was defined as a *p*-value of <0.05. Statistical analyses were performed using JMP version 15.0.0 software (SAS Institute, Cary, NC, United States).

## 3 Results

### 3.1 IL-6R antibody treatment significantly improved muscle weakness in the EAMG mouse model

**Figure 1:**
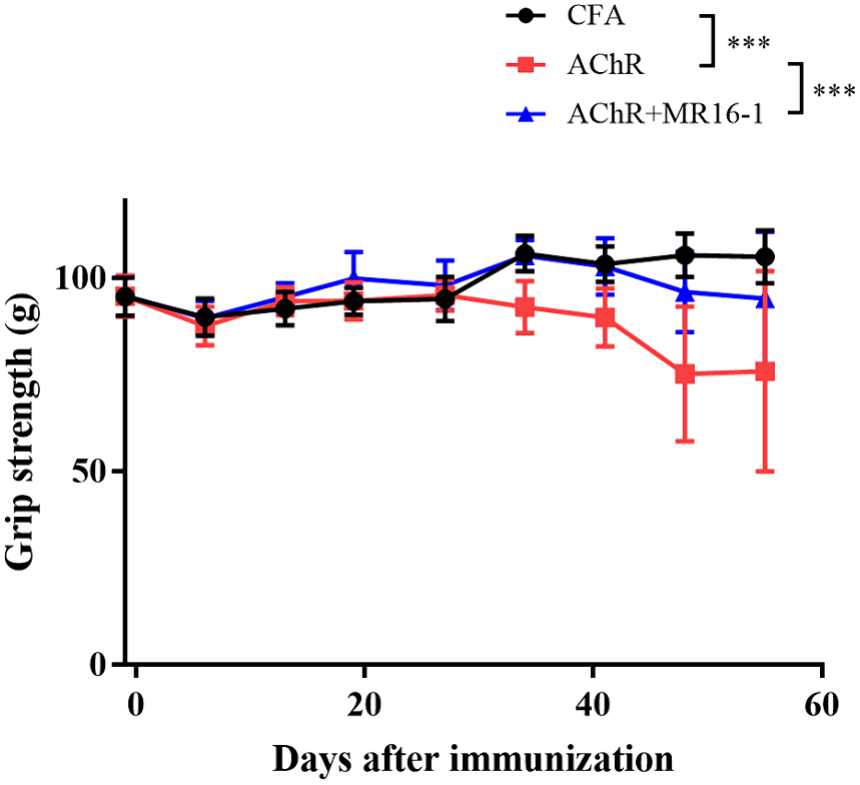
Effect of IL-6 receptor antibody (MR16-1) treatment on muscle weakness in AChR-immunized mice. Grip strength was measured every week in mice from the CFA-treated control group (black circles), AChR-immunized group (red squares), and AChR-immunized + MR16-1-treated group (blue triangles) (*n* = 10 per group). Data are presented as mean ± SD. ***p < 0.001 by two-way ANOVA.

Grip strength was significantly lower in AChR-immunized mice than in CFA-treated control mice. MR16-1 significantly improved muscle weakness in AChR-immunized mice compared with vehicle-treated AChR-immunized mice (Figure 1).

These results indicate that MR16-1 significantly improved muscle weakness in the EAMG mouse model.

### 3.2 IL-6R antibody treatment decreased IgG deposition at the neuromuscular junction

**Figure 2:**
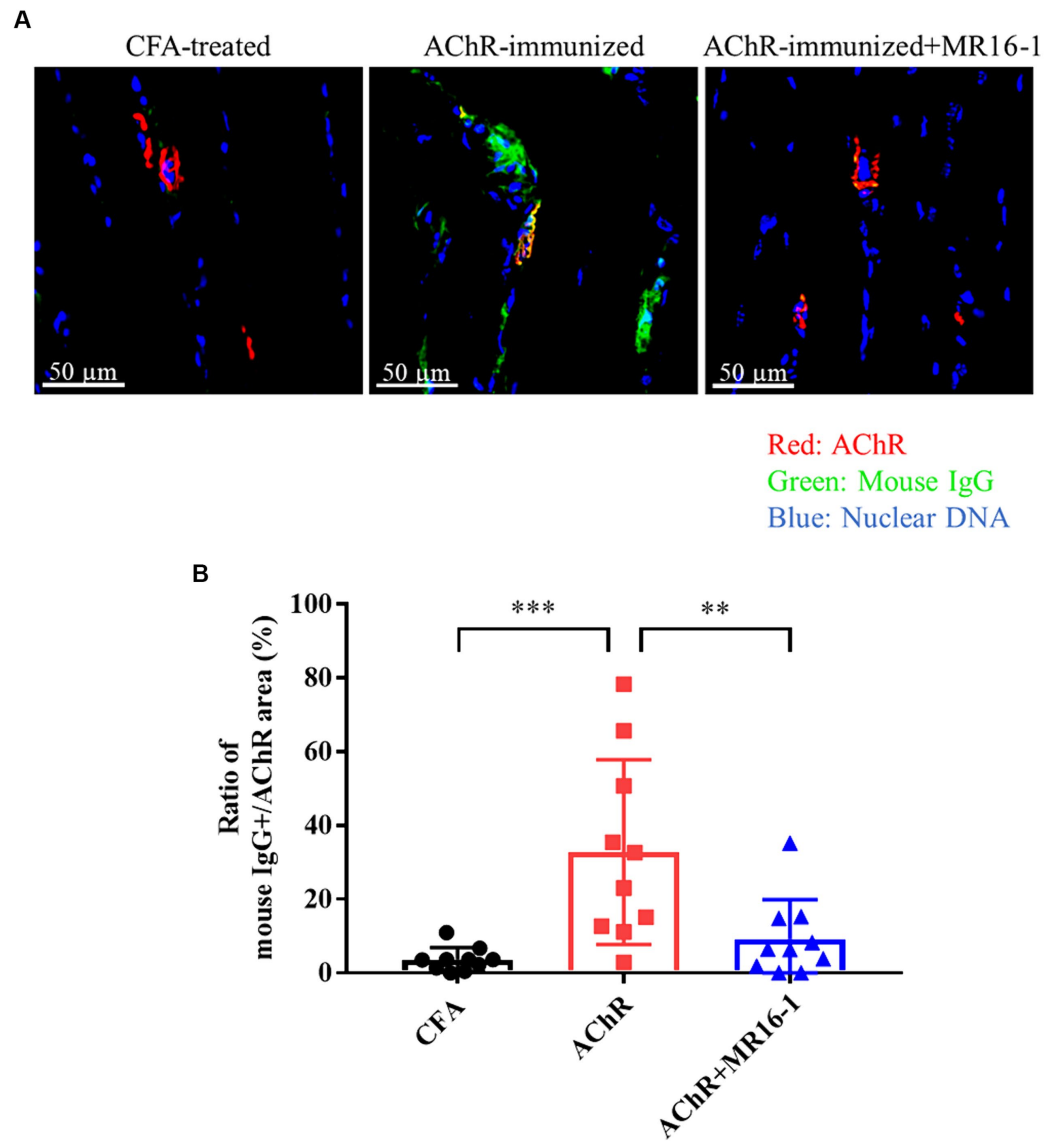
Effect of IL-6 receptor antibody treatment on deposition of anti-AChR IgG in muscle in AChR-immunized mice. **(A)** Co-localization of mouse IgG and AChR in the tibialis anterior muscle was detected by co-staining with anti-mouse IgG (green) and alpha-bungarotoxin (red). Nuclear DNA was stained with DAPI (4′,6-diamidino-2-phenylindole) (blue). **(B)** Mouse IgG-positive area as a percentage of AChR area was quantified by using HALO AI imaging analysis software in CFA-treated control group (black circles), AChR-immunized group (red squares) and, AChR-immunized + MR16-1-treated group (blue triangles) (*n* = 10 per group). Data are presented as mean ± SD. **p < 0.01, ***p < 0.001 by Tukey’s multiple comparison test.

Neuromuscular transmission failure in MG is caused by AChR autoantibodies binding to AChR at the neuromuscular junction (3). Therefore, we investigated whether IL-6R antibody treatment prevented the deposition of AChR autoantibodies in the muscle of EAMG mice. In the tibialis anterior muscle of CFA-treated control mice, there was no co-localization of AChR and mouse IgG (Figure 2A). In AChR-immunized mice, in contrast, co-localization of AChR and mouse IgG was detected, which suggests that AChR autoantibodies were deposited at the neuromuscular junction (Figure 2A). In AChR-immunized mice treated with MR16-1, the area of mouse IgG-positive tissue as a percentage of the area of AChR-positive tissue was lower than that in AChR-immunized mice treated with vehicle alone (Figure 2B).

These results suggest that IL-6R antibody treatment improved the muscle weakness in EAMG mice by decreasing the deposition of AChR autoantibodies in the neuromuscular junction.

### 3.3 IL-6R antibody treatment significantly reduced the levels of AChR autoantibodies in serum

**Figure 3:**
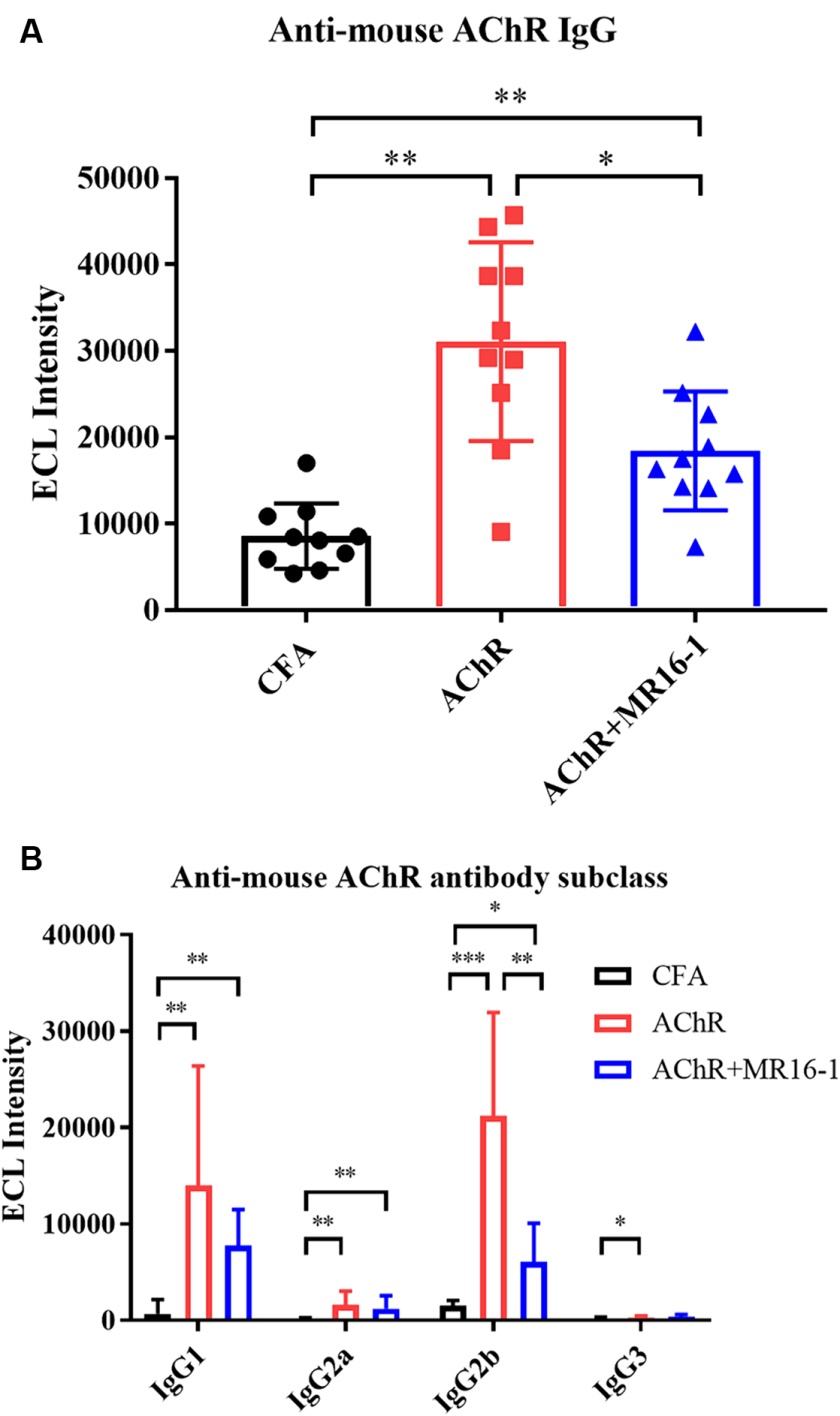
Effects of IL-6 receptor antibody treatment on levels of AChR autoantibodies in serum. Anti-mouse AChR IgG **(A)** and IgG subclasses **(B)** in serum samples from the CFA-treated control group (black circles), AChR-immunized group (red squares), and AChR-immunized + MR16-1-treated group (blue triangles) were measured by ECL (*n* = 10 per group). Data are presented as mean ± SD. *p < 0.05, **p < 0.01, ***p < 0.001 by Steel–Dwass’s multiple comparison test.

Next, we investigated whether IL-6R antibody treatment suppressed the production of AChR autoantibodies. Serum samples were collected at 8 weeks after first inoculation. Levels of AChR autoantibodies measured by ECL were significantly higher in serum from AChR-immunized mice than from CFA-treated control mice. Administration of MR16-1 lowered the levels of AChR autoantibodies in serum of AChR-immunized mice (Figure 3A). Examination of IgG subclasses revealed that MR16-1 lowered the levels of IgG2b subclass in serum of AChR-immunized mice (Figure 3B).

These results indicate that MR16-1 significantly reduced production of AChR autoantibodies in EAMG mice.

### 3.4 IL-6R antibody treatment modulated immune responses in lymph nodes

**Figure 4:**
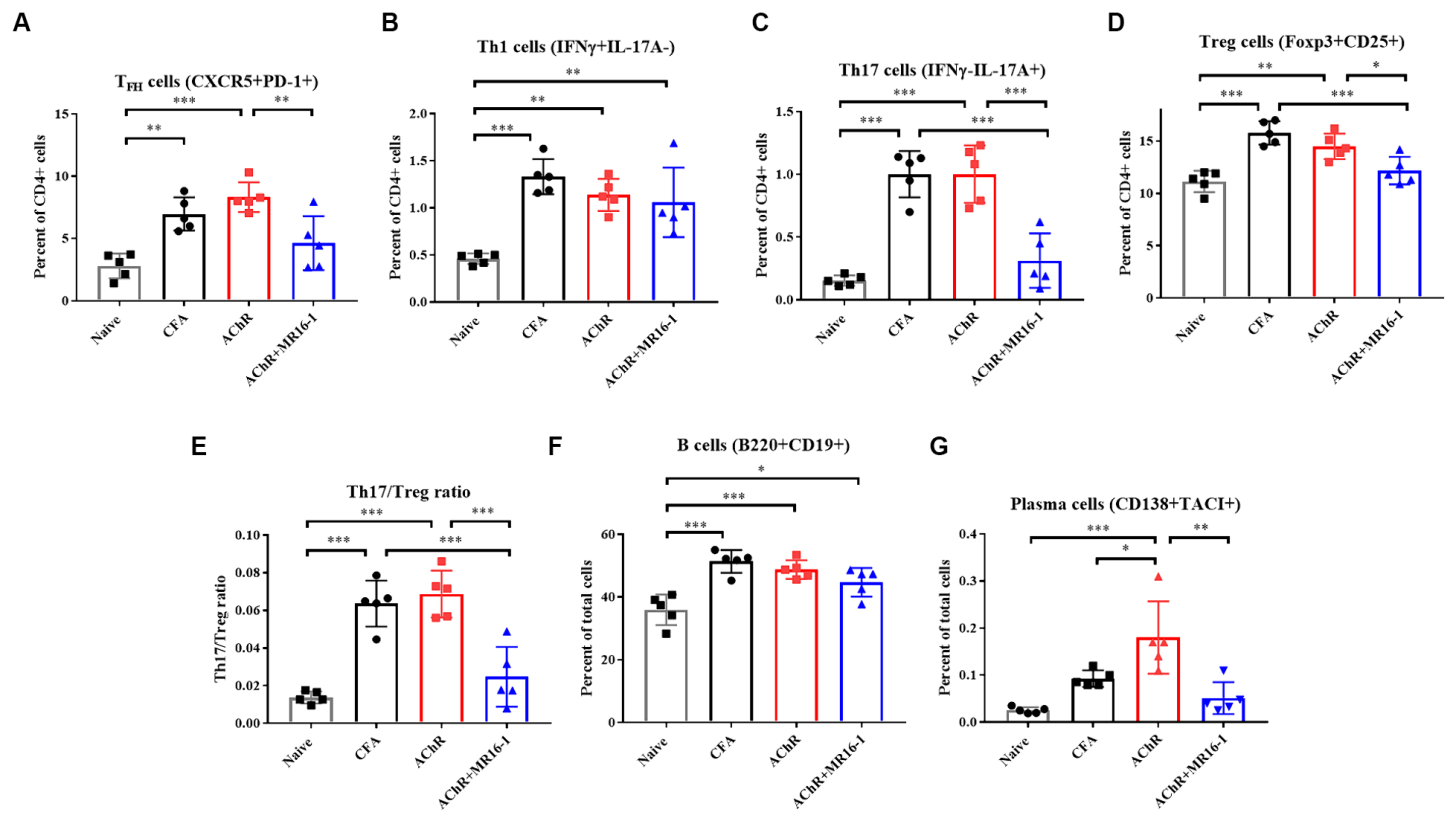
Effects of IL-6 receptor antibody treatment on immunophenotype in lymph nodes. Immunophenotyping of EAMG mouse in lymph nodes **(A–G)** of Naive (black squares), CFA-treated group (black circles), AChR-immunized group (red squares), and AChR-immunized + MR16-1-treated group (blue triangles) (*n* = 5 per group). Data are presented as mean ± SD. *p < 0.05, **p < 0.01, ***p < 0.001 by Tukey’s multiple comparison test.

T_FH_ cells are critical in germinal center formation and antibody production (21). It has been previously reported that the proportion of T_FH_ cells among CD4^+^ T cells is higher in EAMG rats than in control rats (22). In addition, it has been reported that IL-6 is involved in T_FH_ differentiation (10). Therefore, we investigated whether anti-IL-6R antibody suppressed T_FH_ cells related gene expression in lymph nodes. In MR16-1-treated mice, expression of B cell lymphoma 6 (*Bcl6*), a transcription factor for T_FH_ differentiation, and IL-21, a cytokine produced from T_FH_, were lower than AChR-immunized mice. In addition, expression levels of transcription factors for formation of plasma cells, such as *Prdm1*, *Irf4*, and *Xbp1* were also lower in the MR16-1-treated mice (Supplementary Figure S1). Accordingly, we investigated whether the treatment with IL-6R antibody could suppress T_FH_ cells and plasma cells in lymph nodes. At 1 week post the second inoculation, we harvested the inguinal lymph nodes and confirmed the population of T and B cells. In AChR-immunized mice, the percentage of T_FH_, Th1, Th17, and Treg cells among CD4 positive cells were higher than in naive mice (Figures 4A–D). T_FH_ and Th17 cells were lower in MR16-1-treated mice compared with AChR-immunized mice (Figures 4A,C). However, the administration of MR16-1 did not affect Th1 cells (Figure 4B). Although Treg cells were lower in mice treated with MR16-1 compared with AChR-immunized mice, the Th17/Treg ratio also decreased (Figures 4D,E). We further investigated the effect of MR16-1 on B cells and plasma cells. In AChR-immunized mice, the percentage of B cells and plasma cells was higher than in naive mice (Figures 4F,G). The proportion of plasma cells was lowered in MR16-1-treated mice, although there was no difference in the proportion of B cells (Figures 4F,G).

In conclusion, our findings suggest that the administration of MR16-1 effectively reduces the proportion of T_FH_, plasma cells and Th17/Treg balance in the lymph nodes, potentially elucidating a novel mechanism of immune modulation in MG.

## 4 Discussion

In the present study, we provide preclinical evidence for the beneficial effect of IL-6R antibody treatment with respect to the signs and symptoms of MG in EAMG mice. We demonstrated that MR16-1 modulated the immune response and significantly improved muscle weakness.

We discovered that administration of IL-6R antibody decreased T_FH_ cells and plasma cells in lymph nodes and the production of autoantibodies in serum. T_FH_ cells are a subset of the CD4^+^ T cell population that supports the differentiation of antigen-specific B cells into plasma cells (23). It is reported that cT_FH_ cells isolated from MG patients promote antibody production from B cells compared with healthy controls, suggesting that cT_FH_ may be involved in the production of autoantibodies in MG (14, 24). The formation of T_FH_ cells requires interaction with antigen-presenting cells and STAT3-activating cytokines, including IL-6 and IL-21 (23). In fact, T_FH_ cells were not observed in IL-6-deficient mice after lymphocytic choriomeningitis virus infection (10). Thus, our results suggest that IL-6R antibody treatment inhibits autoantibody production via modulating T_FH_ cells. Previous reports have also shown that MG symptoms and autoantibody production are suppressed in IL-6-deficient mice or in rats administered anti-IL-6 antibodies (25, 26). Those reports support the validity of IL-6 as a therapeutic target.

Since IL-6 is a pleiotropic cytokine, blocking IL-6 signaling could have diverse effects on the pathogenesis of MG. In our study, administration of IL-6R antibody decreased Th17 in lymph nodes. Th17 is also a CD4^+^ T cell subset induced by IL-6 and TGFβ signaling, and it plays a pivotal role in autoimmune diseases such as psoriasis, rheumatoid arthritis, and multiple sclerosis (27). In gMG patients, levels of IL-17A in plasma are increased (28) and mRNA expression of IL-17A in peripheral blood mononuclear cells (PBMCs) is also elevated and shows a correlation with AChR autoantibody titer (29). In addition, IL-17 deficient mice showed less symptoms in EAMG mice (30). Therefore, IL-6R antibody treatment may improve muscle weakness by decreasing Th17 in the EAMG mouse model. Furthermore, administration of IL-6R antibody decreased Th17/Treg ratio. Treg cells maintains peripheral tolerance via multiple mode of action and prevents autoimmunity (31). Previous report showed adoptive transfer of Treg cells suppressed clinical symptoms and reduced the levels of AChR autoantibody in EAMG rat (32). Treg cells directly induce plasma cell death and control humoral autoimmunity (33). Therefore, IL-6R antibody treatment may improve muscle weakness and suppress autoantibody production via modulating Treg cells.

There are several limitations to this study. We suggest that IL-6R inhibition decreased T_FH_ and Th17 cells. However, our results cannot mention the mechanisms of T cell suppression. Especially, IL-6 signaling plays a pivotal role in maintaining T_FH_ cells by ensuring IL-2 hyporesponsiveness through the inhibition of IL-2Rβ up-regulation (34). Therefore, IL-6 is involved in both the differentiation and maintenance of T_FH_ cells, but the effect of IL-6R antibody has not been investigated.

Our study suggested that one of the mechanisms through which IL-6R antibody treatment may improve muscle weakness is by targeting T_FH_ cells. In MG patients, the percentage of circulating cT_FH_ cells in the population of CD4^+^ T cells is increased and is positively correlated with quantitative MG score (14). It has also been reported that in MG patients with diabetes mellitus, hyperglycemia induces further increase and activation of cT_FH_, thereby promoting plasmablast differentiation and IgG secretion (35). Therefore, inhibition of IL-6 signaling may decrease cT_FH_ cells and IgG secretion in MG patients.

In conclusion, we found that IL-6R antibody treatment significantly improved muscle weakness in the EAMG mouse model. Additionally, production of AChR autoantibodies and T_FH_, Th17 and plasma cell in lymph nodes were also decreased. Thus, targeting IL-6R may provide a novel and effective therapeutic strategy for the treatment of MG.

## Data availability statement

The original contributions presented in the study are included in the article/Supplementary material, further inquiries can be directed to the corresponding author.

## Ethics statement

The animal study was approved by Institutional Animal Care and Use Committee at Chugai Pharmaceutical Co., Ltd. The study was conducted in accordance with the local legislation and institutional requirements.

## Author contributions

SM: Visualization, Project administration, Writing – original draft, Validation, Methodology, Investigation, Formal analysis, Conceptualization. KS: Writing – original draft, Supervision, Project administration, Investigation, Conceptualization. SO: Investigation, Writing – review & editing, Formal analysis. YK: Investigation, Writing – review & editing. MB: Investigation, Writing – review & editing. MK: Investigation, Writing – review & editing. HT-S: Investigation, Writing – review & editing. KY: Investigation, Writing – review & editing. YM: Writing – review & editing, Supervision. MN-S: Writing – review & editing, Supervision.

## Supplementary material

Figure S1 Effect of IL-6 receptor antibody treatment on gene expression in lymph nodes. mRNA expression of genes involved in immune response of antibody production in lymph nodes of CFA-treated group (black circles), AChR-immunized group (red squares), and AChR-immunized + MR16-1-treated group (blue triangles) (*n* = 10 per group). mRNA expression levels of *Il21*, *Bcl6*, *Prdm1*, *Irf4*, and *Xbp1* were measured by qRT-PCR. Data are presented as mean ± SD. ***p* < 0.01, ****p* < 0.001 by Tukey’s multiple comparison test.
