# Supplementary figures and images for: IL-6 receptor antibody treatment improves muscle weakness in experimental autoimmune myasthenia gravis mouse model
Source: Front Neurol. 2024 May 1;15:1356300. doi: 10.3389/fneur.2024.1356300 (PMC11094227; doi:10.3389/fneur.2024.1356300)

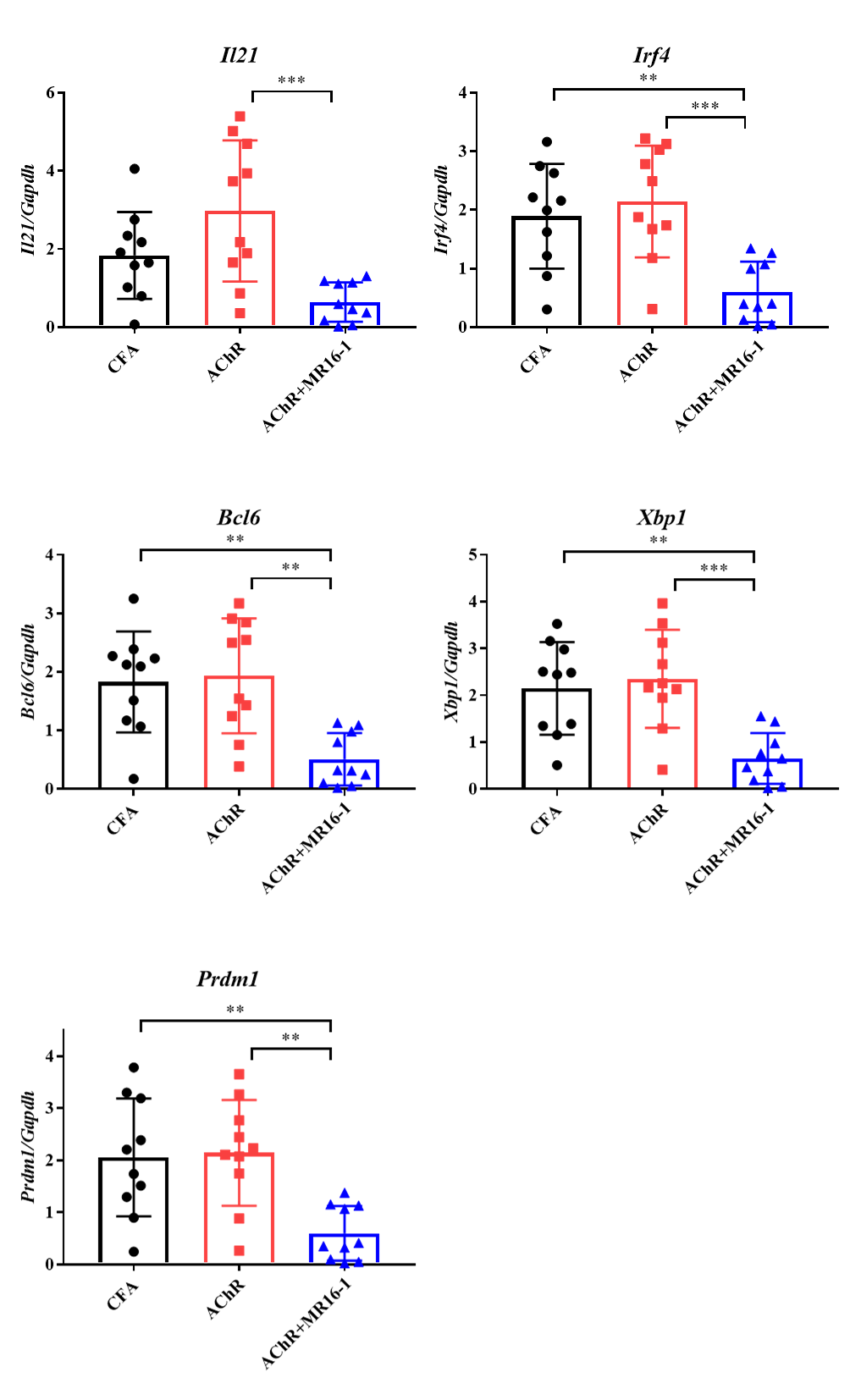

Supplement: Figure S1 — Effect of IL-6 receptor antibody treatment on gene expression in lymph nodes. mRNA expression of genes involved in immune response of antibody production in lymph nodes of CFA-treated group (black circles), AChR-immunized group (red squares), and AChR-immunized + MR16-1-treated group (blue triangles) (n = 10 per group). mRNA expression levels of Il21, Bcl6, Prdm1, Irf4, and Xbp1 were measured by qRT-PCR. Data are presented as mean ± SD. **p < 0.01, ***p < 0.001 by Tukey’s multiple comparison test. [file Image_1.TIF]
